# Potential Diagnostic Approaches for Prediction of Therapeutic Responses in Immune Thrombocytopenia

**DOI:** 10.3390/jcm10153403

**Published:** 2021-07-30

**Authors:** Anne-Tess C. Jolink, Vivianne S. Nelson, Martin R. Schipperus, Sufia N. Amini, Gestur Vidarsson, C. Ellen van der Schoot, Leendert Porcelijn, Masja de Haas, Rick Kapur

**Affiliations:** 1 Sanquin Research, Department of Experimental Immunohematology, Landsteiner Laboratory, Amsterdam UMC, University of Amsterdam, 1066 CX Amsterdam, The Netherlands; atcjolink@gmail.com (A.-T.C.J.); g.vidarsson@sanquin.nl (G.V.); e.vanderschoot@sanquin.nl (C.E.v.d.S.); 2 Sanquin, Department of Transfusion Medicine, 1066 CX Amsterdam, The Netherlands; v.nelson@hagaziekenhuis.nl (V.S.N.); m.r.schipperus@umcg.nl (M.R.S.); s.amini@hagaziekenhuis.nl (S.N.A.); 3 Department of Hematology, University Medical Centre Groningen (UMCG), 9713 GZ Groningen, The Netherlands; 4 Sanquin Diagnostic Services, Department of Immunohematology Diagnostics, 1066 CX Amsterdam, The Netherlands; l.porcelijn@sanquin.nl (L.P.); m.dehaas@sanquin.nl (M.d.H.); 5 Leiden University Medical Center, Department of Hematology, 2333 ZA Leiden, The Netherlands

**Keywords:** immune thrombocytopenia (ITP), therapeutic responses, therapy, therapy prediction, diagnostics

## Abstract

Immune thrombocytopenia (ITP) is an autoimmune bleeding disorder in which, via unresolved mechanisms, platelets and megakaryocytes (MKs) are targeted by autoantibodies and/or T cells resulting in increased platelet destruction and impairment of MK function. Over the years, several therapeutic modalities have become available for ITP, however, therapeutic management has proven to be very challenging in several cases. Patients refractory to treatment can develop a clinically worsening disease course, treatment-induced toxicities and are predisposed to development of potentially life-endangering bleedings. It is therefore of critical importance to timely identify potential refractory patients, for which novel diagnostic approaches are urgently needed in order to monitor and predict specific therapeutic responses. In this paper, we propose promising diagnostic investigations into immune functions and characteristics in ITP, which may potentially be exploited to help predict platelet count responses and thereby distinguish therapeutic responders from non-responders. This importantly includes analysis of T cell homeostasis, which generally appears to be disturbed in ITP due to decreased and/or dysfunctional T regulatory cells (Tregs) leading to loss of immune tolerance and initiation/perpetuation of ITP, and this may be normalized by several therapeutic modalities. Additional avenues to explore in possible prediction of therapeutic responses include examination of platelet surface sialic acids, platelet apoptosis, monocyte surface markers, B regulatory cells and platelet microparticles. Initial studies have started evaluating these markers in relation to response to various treatments including glucocorticosteroids (GCs), intravenous immunoglobulins (IVIg) and/or thrombopoietin receptor agonists (TPO-RA), however, further studies are highly warranted. The systematic molecular analysis of a broad panel of immune functions may ultimately help guide and improve personalized therapeutic management in ITP.

## 1. Introduction

Immune thrombocytopenia (ITP) is an acquired hematological autoimmune bleeding disorder characterized by an isolated thrombocytopenia (peripheral blood platelet count < 100 × 10^9^/L) and may clinically present as petechiae, purpura, mucosal bleedings, intracranial hemorrhages (~0.2% of the cases), with a reduced health-related quality of life (HRQoL) [1]. Clinically, various disease phases can be distinguished: newly diagnosed, persistent (symptoms lasting between 3 and 12 months) and chronic ITP (symptoms remaining present after 12 months) [1]. Therapeutic management approaches include initial treatments such as corticosteroids, intravenous immunoglobulin (IVIg) or anti-D, and subsequent treatment options include rituximab, thrombopoietin receptor agonists (TPO-RA) and splenectomy [2,3]. Unfortunately, a significant proportion of ITP patients relapse after treatment. A survey of several studies suggests that almost two-thirds of patients have at least a partial immediate response to corticosteroids and three-quarters have some response to IVIg or anti-D [4]. It has been estimated that approximately 40–60% of newly diagnosed adult ITP patients have a sustained response to initial treatment with standard first-line therapies (standard-dose corticosteroids, IVIg, anti-D) after 6 months [5]. Only a quarter of adults with newly diagnosed ITP, however, remains relapse-free beyond one year [5]. Noteworthy, these results on long term response rates to first-line therapy show a high variability of 8–43% which may reflect the differences in ITP patient population, duration and dose of treatment and in definitions of platelet response. Although prospective data on long-term therapy response is not readily available, retrospective studies suggest that the response rate after 2 to 10 years decreases significantly to 20–30% [5]. Approximately 5–10% of patients diagnosed with ITP are considered to be unresponsive to any initial therapy [4,6], i.e., refractory ITP. Refractory ITP patients do not only respond poorly to different therapies, but they also develop worsening disease, medication-induced toxicities and are predisposed to develop bleedings [7]. In addition, multi-refractory ITP is a potentially life-threatening disorder due to risk of significant bleeding events and high mortality (observed in 5/30 chronic ITP patients (14%)) and morbidity (observed in 9–15/30 chronic ITP patients (24–60%); varying from platelet transfusions to admission to an intensive care unit) [8]. Timely identification of refractory patients is therefore of critical importance, for which novel diagnostic tools that can monitor and predict therapeutic responses to specific treatment modalities are highly warranted. Recent insights into this will be discussed in the current paper, and how they may serve as a much-needed stepping stone for the development of novel diagnostic assays which may aid in managing treatment approaches in ITP.

## 2. Pathophysiology of ITP

The pathogenesis of ITP is complex and multifactorial. It is recognized, through incompletely understood mechanisms, that platelet destruction occurs via platelet autoantibodies and/or T cell mediated platelet destruction and/or impaired platelet production by bone marrow (BM)-residing megakaryocytes (MKs) [1,9]. The principal mechanism of platelet clearance occurs via IgG-platelet autoantibodies directed against platelet surface glycoproteins, mostly GPIIb/IIIα (45–71%), GPIb-IX (64–68%) and GPV (65–83%) [10,11]. In adults, autoantibodies of the IgG class are predominantly found and more rarely of the IgM or IgA class [12], whereas in children often IgM class autoantibodies are present [13]. Autoantibodies bound to platelets are subsequently recognized by monocytes/macrophages bearing Fcγ receptors (FcγRs), resulting in phagocytic breakdown in the spleen and liver [1,14]. Interestingly, it has recently been demonstrated that FcγRI and FcγRIII on splenic macrophages primarily mediate anti-GPIIb/IIIα opsonized platelet clearance in vitro [15]. C-reactive protein (CRP), an acute phase protein upregulated during acute infections and inflammation, was shown to functionally enhance anti-platelet antibody-mediated phagocytosis in vitro and antibody-mediated platelet clearance in vivo [16]. In addition, CRP was found to be elevated in newly diagnosed ITP patients, and IVIg treatment led to a decrease in CRP levels, elevated platelet counts and a decreased bleeding severity [16]. Importantly, increased CRP levels at diagnosis predicted slower platelet count recovery three months after diagnosis [16]. Furthermore, autoantibodies can also target MKs in the (BM) leading to defective megakaryopoiesis [1]. In approximately 20% of patients that are suspected to have ITP, however, no circulating autoantibodies can be detected [17], which supports the involvement of other mechanisms of platelet clearance like Fc-independent platelet clearance through hepatic Ashwell-Morrel receptors [18]. Indeed, cytotoxic CD8+ T cells have been shown to be able to directly lyse platelets in active ITP [19]. Cytotoxic CD8+ T cells have been demonstrated to be activated in ITP, inducing MK apoptosis and dysregulation of BM homeostasis leading to defective megakaryopoiesis and thrombopoiesis [20]. An important feature in the pathogenesis of ITP is the apparent loss of immune tolerance due to an imbalance in T-cell homeostasis, which is signified by an impairment of CD4+ CD25+ FoxP3+ T regulatory cells (Tregs). Treg impairment in ITP is characterized by a reduction in numbers and/or a functional defect in the immunosuppressive function [1,9]. This is accompanied by an expansion in T-helper (Th)1 and Th17 cells, which produce IFN-γ and IL-17 respectively [1,9]. Tregs maintain immune tolerance by inhibiting immune responses which culminate in the immune destruction of platelets. The Treg/Th imbalance thus allows the occurrence of immune-mediated platelet destruction. Therefore, Tregs, but also dendritic cells (DCs), are likely the initiators and perpetuators of ITP [9,21]. It also has been suggested that myeloid derived suppressor cells (MDSCs, CD11b+ CD33+ HLA-DRlow), cells of myeloid origin with the ability to suppress T cell responses, are also impaired in number and in suppressive functions in peripheral blood and spleens of patients with ITP [22]. These data suggest that dysregulation in Tregs, DCs and MDSCs may perhaps play a combinational role in the loss of immune tolerance in ITP. Additionally, a lower number of B regulatory cells (Bregs, CD19+ CD24hi CD38hi) and higher number of CD19+ CD24+ CD38- B memory cells (Bmems) has been observed in peripheral blood of newly diagnosed pediatric patients with ITP [23]. Bregs produce interleukin (IL)-10 and control and maintain Tregs by stimulating their differentiation and/or recruitment [24]. Moreover, the dysregulated B cell compartment observed in patients with ITP was subsequently shown to lead to a reduced B cell suppressive activity with less dampening of monocyte activation in patients with low platelet counts [24]. Overall, multiple cell types are involved in the pathogenesis of ITP [1,25]. In the last decade, it has become increasingly clear that platelets possess important non-hemostatic immune functions and characteristics [26,27,28,29,30], for which we hypothesize that they may be impaired in the pathophysiology of ITP. Platelets are both capable of recognizing and targeting pathogens, and of communicating with distinct target cells in order to, for example, stimulate B cell or DC differentiation, activate neutrophils and modulate T cell responses, e.g., [26,27,30]. This cellular communication may also occur via secretion of cytokines or chemokines, or by shedding of platelet microparticles (PMPs), which may for instance contain chemokines, cytokines, mitochondria, lipids, transcription factors and RNA [26,27,30]. 

Due to the complexity and heterogeneity of the ITP pathophysiology as well as the heterogeneity of different disease phases in ITP, the therapeutic management remains challenging. Therapy resistance, relapse and/or refractoriness are frequently encountered, and the responsible mechanisms are likely multifactorial and have not been fully resolved. For instance, 60% of ITP patients fail to respond to rituximab [31]. It was suggested that lack of platelet autoantibodies may be correlated to a non-response to rituximab [32]. Furthermore, two B cell populations (rituximab-resistant memory B cells, and autoreactive B cells never exposed to rituximab) were recently suggested to contribute to relapses upon rituximab treatment [33]. In addition, rituximab non-responders were found to have splenic CD8+ T cell activation which may contribute to platelet destruction [34]. Overall, it will be important to decipher the heterogeneity in the pathophysiology of ITP, in order to predict therapeutic responses, which is an unmet clinical need in the management of ITP patients. We hypothesize that immune functions and characteristics of platelets are likely impaired in ITP, and alterations in these markers induced by therapies in ITP may be exploited for the design of novel diagnostic approaches which may help predict responders and non-responders to specific treatment modalities.

## 3. Current Diagnostics for ITP

Immune thrombocytopenia is a diagnosis of exclusion where thrombocytopenia is confirmed through analysis of complete blood counts and exclusion of the presence of platelet morphology abnormalities (suggestive of e.g., hereditary platelet disorders or myelodysplasia) using peripheral blood smear [35]. Other causes of thrombocytopenia, such as a Hepatitis C Virus and Human Immunodeficiency Virus, must be evaluated as they may explain the observed thrombocytopenia and treating the underlying disorder might improve the platelet count [2,35]. In addition, the majority of the international ITP expert panel recommends to also test for H. Pylori in adult patients in the appropriate geographical setting [2]. In some of these cases, molecular mimicry may have induced development of GP-reactive antibodies [36]. GP-specific antibody testing may be of potential utility for ITP diagnosis according to the updated international consensus report on investigation and management of primary ITP [2]. As autoantibody-mediated platelet destruction is considered a principal mechanism in ITP, platelet GP-specific autoantibody detection may be considered, at least from a diagnosis-supporting perspective, as a valuable contribution for ITP diagnosis [17]. Several methods have been developed for platelet autoantibody detection, including enzyme-linked immunosorbent assay (ELISA)-based monoclonal antibody immobilization of platelet antigen (MAIPA) assay [17,37,38]. In comparison with (commercially available) GP-specific autoantibody detection assays using GP pre-coated microtiter plates or beads, the MAIPA assay has the advantage that human antibodies can bind to epitopes on platelet GPs in their natural configuration on the membrane. In addition, the MAIPA has a good specificity (>95%) and reasonable sensitivity (80%) and is therefore limited to be a ‘rule in’ tests for ITP [17,39]. Detected autoantibodies, however, may not necessarily indicate disease severity since antibody-mediated phagocytic potency and biological activity are not tested. Notably, however, is the fact that despite that quantitative and/or qualitative dysregulation of Tregs is likely a critical factor responsible for the disrupted immune balance in ITP, Treg analyses is not yet diagnostically performed. Regarding therapeutic responses, it has been suggested that therapeutically restoring immune tolerance in ITP by reversing the Treg impairment and suppressing Th1 and Th17 cells may be important for a sustained therapeutic response [1,9]. Additionally, other immune functions and characteristics may be valuable to probe therapeutic responses. In this paper, we advocate to broaden our scope and potentially expand our diagnostic approach by analyzing immune characteristics and functions in ITP, particularly T cell homeostasis, but also platelet surface sialic acids, platelet apoptosis, monocyte surface markers, B regulatory cells and platelet microparticles. This may potentially help us to better understand and to possibly predict specific therapeutic responses in ITP. This paper is thus primarily meant to stimulate the diagnostic field regarding therapy resistance in ITP, for which we provide suggestions for several investigational studies. The routine diagnosis and management of ITP are briefly discussed; however, this is not the scope of this manuscript. For more information on this, we refer the readers to the international consensus report and American Society of Hematology guidelines [2,3].

## 4. T Cell Homeostasis 

CD4+ Tregs are a subgroup of T cells with immunomodulatory and immunosuppressive function. More than two decades ago, CD4+ Tregs have been identified as critical immunosuppressive cells [40] which play an important role in maintaining immune homeostasis [41] and preventing autoimmunity [42], including in autoimmune disorders like rheumatoid arthritis (RA) [43], systemic lupus erythematosus (SLE) [44] and juvenile idiopathic arthritis [45]. In addition, Tregs also appear to be important immunosuppressive cells in alloimmune responses such as in transfusion-related acute lung injury (TRALI) [46]. In ITP, Tregs are also important as they maintain immune tolerance by inhibiting pathogenic T cell and antibody responses towards platelets [1,9]. Patients with newly diagnosed and chronic ITP showed an impairment in Tregs characterized by a reduction in number and/or a functional defect in immunosuppressive function at disease onset, e.g., [47,48]. This quantitative and qualitative impairment in Tregs has been shown to be paralleled by an increase in CD3+ CD4+ IL-17-producing Th17 cells [1,49]. Likewise, patients with ITP show a concordant increased cytokine imbalance towards IFN-γ and IL-2, indicating a shift towards Th1 cells [50]. The dysregulated T cell homeostasis signified by an imbalance in Th1/Th17 vs. Tregs is correlated with disease activity in ITP [21], and therefore appears to be an important feature which may be responsible for the loss of immune tolerance and onset of the disease. When platelet counts increase in response to treatment, the Treg numbers and their immunosuppressive activity normalize [1,21]. Various therapeutic regimens have demonstrated efficacy in ITP in relation to an increase in the number of Tregs, such as IVIg [51], glucocorticosteroids (GCs) [51,52], dexamethasone [53], TPO-RA [54] and low-dose decitabine [55]. Low-dose decitabine, an antimetabolite and demethylation agent normally prescribed in myelodysplastic syndrome (MDS), was recently shown to increase platelet counts in ITP by improving Treg function and quantity with additional suppression of Th1 and Th17 cells [55]. Besides, it has been demonstrated in multiple studies that Treg normalization is associated with an increase in plasma transforming growth factor beta (TGF-β) and IL-10 levels [21]. Moreover, Manzano and colleagues compared several immune characteristics in ITP related to therapy response [54]. The groups that were investigated included 1) healthy controls (HCs; *n* = 104) 2) untreated ITP patients ((UT-ITP; *n* = 28), patients that went into remission after a period of thrombocytopenia lasting for more than 12 months and who did not need treatment for at least 6 months prior to enrollment 3) ITP patients responding to TPO receptor agonists (TPO-RA; *n* = 36) with a platelet count > 30 × 10^9^/l and at least a two-fold increase from baseline platelet count and absence of bleeding and 4) a group of non-responding ITP patients (NR-ITP; *n* = 14) that did not respond to first- and second line therapies [54]. They observed that UT-ITP and NR-ITP patients had a low number of Tregs in whole blood compared to HCs, indicating that a low number of Tregs is present in active and non-responding ITP. Moreover, it was observed that patients treated with TPO-RA had a higher number of Tregs than NR-ITP, suggesting that normalization of Tregs is indicative of successful treatment with TPO-RA [54]. Although CD4+ Tregs have been associated with the pathophysiology of ITP by a plethora of studies, CD8+ CD25str+ Tregs can also play an important role in immune modulation [56]. CD8+ Tregs yield the ability to activate autoreactive T cells, cause proliferation of autoreactive T cells and inhibit the release of pro-inflammatory cytokines through expression of high levels of FoxP3, as well as GC induced tumor necrosis factor (TNF) receptor, TNF receptor type 2 and CTLA-4 [56]. A recent study in newly diagnosed adult ITP patients (*n* = 55), who did not receive treatment in at least 3 months prior to enrollment, demonstrated that in the GC-sensitive group the levels of CD8+ CD25str+ Tregs were significantly higher than in the GC-insensitive group, while no obvious changes were observed for CD4+ Tregs [57]. This suggested that CD8+ Tregs cells may possibly be predictive for GC sensitivity [57], however, further validation is warranted. Furthermore, differentially skewed CD4+/CD8+ ratio combined with a higher absolute number of CD19+ B lymphocytes has been observed in newly diagnosed adult ITP patients that responded to monotherapy with corticosteroids or corticosteroids in combination with IVIg compared to the non-responder group [51]. In summary, although different therapies may have different working mechanisms, the restoration in the defective CD4+ Treg compartment in responding ITP patients appears to be a central feature induced by multiple therapies, making CD4+ Treg analysis an attractive approach for monitoring and possibly predicting specific therapeutic responses.

## 5. Platelet Surface Sialic Acids

Apart from the classical antibody Fc-FcγR-dependent platelet phagocytosis by macrophages, it has been suggested that Fc-independent platelet clearance may also be an important mechanism in ITP, which may occur via antibody-mediated loss of sialic acid from platelet glycoproteins, through hepatic Ashwell-Morell receptors [18]. It was demonstrated that anti-platelet GPIbα-antibodies, but not GPIIb/IIIα-antibodies, induced platelet activation, Neuraminidase 1 (Neu1) translocation and desialylation in a murine model of passive (antibody-induced) ITP [18]. Other studies confirmed the positive correlation between GPIb/IX-antibodies and platelet desialylation, supporting a mainly GPIbα-driven FcγR-independent mechanism of platelet clearance in ITP [58,59]. In contrast, it was recently found that ITP antibody-induced desialylation of platelets was not GPIb-specific, as desialylation induced by anti-GPIIb/IIIα antibodies was even higher than desialylation induced by anti-GPIbα antibodies [60]. Interestingly, another study also observed that both anti-GPIb/IX and anti-GPIIb/IIIα could cause platelet surface-desialylation in ITP patients (*n* = 51) [61]. Among all patients, platelet-desialylation was observed in 26/51 (51%) patients, and more frequently in the presence of GPIIb/IIIα antibodies (15/26 (58%) [61]. Therefore, it appears that desialylation may be caused by both anti-GP GPIb/IX and anti-GPIIb/IIIα. Furthermore, it was demonstrated that autoantibody-mediated desialylation impaired platelet adhesion and megakaryocyte differentiation [61]. Additionally, it was found that autoantibodies inducing desialylation were associated with lower platelet counts and a higher bleeding frequency in ITP patients [61]. Interestingly, Manzano and colleagues investigated the degree of platelet desialylation to therapy response in ITP and observed that NR-ITP patients have lower levels of platelet GP sialic acid compared to healthy controls and to patients treated with TPO-RA [54]. These findings endorse earlier research that also showed a correlation between platelet desialylation and response to first line treatment (corticosteroids and IVIg), with non-responders having a higher degree of platelet desialylation [6]. The importance of loss of sialic acid in ITP and its effect in therapy response has been recently supported by case studies that administered oseltamivir phosphate, a sialidase inhibitor normally prescribed for influenza, to two patients with primary ITP, one chronic pediatric ITP patient and one adult chronic ITP patient [62,63]. This resulted in elevated platelet counts independent of their influenza disease course. Another study confirmed these findings by showing that ITP patients (*n* = 10) with either newly diagnosed (*n* = 1), persistent (*n* = 2) or chronic ITP (*n* = 7), not responding to GCs, IVIg, splenectomy or at least one administration of TPO-RA (multi-refractory patients), responded to oseltamivir with increasing platelet counts (enhanced response with additional treatment with TPO-RA) [64]. These studies support the notion that pharmacological inhibition of platelet glycoprotein desialylation may be able to normalize platelet counts in ITP. Finally, another remarkable finding was the observation of an inverse correlation between Treg counts and loss of platelet surface sialic acids [54]. This observation suggests a possible direct link between loss of platelet surface sialic acids and cellular immune responses in ITP [65]. In regard to that, it was shown that cytotoxic CD8+ T cells induced platelet desialylation, Neu1 expression on the platelet surface (apoptotic opsonization) and phagocytosis by hepatocytes in vitro [66], suggesting that besides Tregs other cellular immune cells may also be involved in the regulation of platelet surface desialylation. All together, these data suggests that low Treg counts and a high degree of platelet desialylation may be indicative for non-responding ITP patients. In addition, a higher degree of platelet surface sialylation may be predictive of response to TPO-RA, corticosteroids and IVIg.

## 6. Platelet Apoptosis

Apoptosis is a form of caspase-mediated programmed cell death characterized by a variety of morphological changes such as expression of apoptotic makers on the outer membrane of the cell, initiation of the caspase cascade and activation of the mitochondrial permeability transition [67]. Apoptosis is one of the mechanisms to eliminate platelets from the circulation [68]. One of the most important morphological changes during apoptosis of platelets is the translocation of membrane phosphatidylserine (PS) from the inner to the outer leaflet of the platelet membrane, leading to exposure of PS to the extracellular space [69]. A study in pediatric patients with newly diagnosed ITP (*n* = 20) found increased proportions of platelets with activated caspase-3, -8 and -9, PS exposure, and a depolarized inner membrane potential (ΔΨm) [70]. Additionally, it was found that the extent of PS exposure and activation of caspase-3, -8 and -9 could be normalized by IVIg treatment and was correlated with an increased platelet count. More recently, this increase in the expression of apoptotic markers, including PS exposure and ΔΨm depolarization, was confirmed in adult patients with chronic (lasting > 12 months) ITP (*n* = 40) compared to healthy controls (*n* = 40) [69]. Unfortunately, whether therapy had any influence on these observations in chronic ITP patients was not investigated. In contrast to the amelioration of apoptotic factors observed after treatment with IVIg [70], another study reported that patients treated with TPO-RA (*n* = 42) had increased apoptotic activity, including increased PS exposure and caspase-3, -7, -8 and -9 compared to untreated patients (*n* = 40) [71]. This was in line with the study by Manzano et al. that also reported an increased caspase-3, -7, -8 and -9 activity in platelets of both TPO-RA treated and NR-ITP patients [54], indicating that unlike for IVIg, the TPO-RA response cannot be monitored or predicted through analysis of platelet apoptosis. Previous research suggested desialylation to be associated with apoptosis and phagocytosis of platelets in patients with prolonged isolated thrombocytopenia after allo-hematopoietic stem cell transplantation [72], however, recent studies suggest that loss of platelet surface sialic acid and platelet apoptosis are not directly related in ITP [54,61]. In sum, it appears that monitoring IVIg response through analysis of platelet apoptosis, in contrast to the response to TPO-RA, may have potential to predict the therapeutic response in ITP. 

## 7. Monocyte Surface Markers 

Monocytes (MCs) play a key role in shaping T cell responses through processes of antigen presentation and cytokine production [73]. An imbalance in T cell homeostasis is a key process in the development of ITP. CD16+ monocytes have been described to promote Th1 development, and to negatively regulate IL-17 and Treg induction in ITP [74]. In addition, antibody-opsonized platelets are presumably phagocytosed by FcγR-expressing monocytes and macrophages in the spleen and liver in ITP [1]. The role of MCs in relation to therapy responses in ITP has not been widely studied. A recent study by Williams et al. examined MC subsets and their cell surface phenotype in peripheral blood mononuclear cells isolated from untreated, newly diagnosed ITP patients with a platelet count <30 × 10^9^/L (*n* = 11) before and after two weeks of in vivo GC treatment [75]. It was found that intermediate (I)-MCs (CD14++ CD16+) were increased in newly diagnosed, untreated ITP patients, with an enhanced I-MC cell surface expression of pro-inflammatory markers CD64 and CD80 compared with healthy controls (HCs; *n* = 10). Following GC treatment, the proportion of I-MCs reduced and displayed enhanced anti-inflammatory markers CD206 and CD163 [75]. Hence, this data suggests a switch from pro-inflammatory to anti-inflammatory phenotypes in I-MC subsets of newly diagnosed ITP after response to GC treatment. Notably, Manzano et al. did not observe increased I-MCs in their untreated cohort of ITP patients [54]. This could be related to their cohort suffering from ITP for a minimum of 6 months while Williams and colleagues focused on newly diagnosed ITP with a platelet count of less than 30 x 10^9^/l. In conclusion, monitoring and phenotyping I-MC could be of value in assessing GC therapy efficacy in newly diagnosed ITP patients and may aid in therapeutic management decisions. 

## 8. B regulatory Cells (Bregs)

B cells take part in immune responses by producing antibodies and cytokines and by antigen-presentation to T cells. In ITP, a lower number of CD19+ CD24hi CD38hi B regulatory cells (Bregs) and higher number of CD19+ CD24+ CD38- Bmems has been observed [23,24]. Bregs produce IL-10 and control and maintain Treg differentiation and/or recruitment to sites of inflammation [24]. Functionally, Bregs of ITP patients were found to be impaired in their ability to inhibit monocyte activation (assessed via monocyte TNF-α expression) [24]. Interestingly, analyses of Breg counts in 5 non-splenectomized ITP patients after TPO-RA treatment, which elevated platelet counts, showed increased Breg frequencies compared to those before treatment [24]. Furthermore, another study also found elevated Breg counts in chronic ITP patients responding to TPO-RA, in contrast to UT-ITP, NR-ITP patients and HCs [54]. This study did not evaluate Breg function before and after treatment with TPO-RA. These studies indicate that the quantitative (and perhaps the functional) defect in Bregs can be restored in chronic ITP patients responding to TPO-RA. This indicates that, at least in chronic ITP patients, an increase in Breg numbers (in combination with increased functionality with respect to dampening monocyte activation) could potentially be of predictive value for the response to TPO-RA. 

## 9. Platelet Microparticles (PMPs) 

Platelet microparticles (PMPs) are small (0.1–1 μm), heterogeneous vesicles produced upon activation of platelets and released from the cellular plasma membrane [76,77]. PMPs facilitate communication to adjacent or distant cells [78], by transporting and delivering different kinds of cargo, such as chemokines, surface receptors, nucleic acids, autoantigens, transcription factors, potent lipid mediators, functional enzymes and mitochondria [26]. Using flow cytometric methodologies, PMPs have been observed in different inflammatory conditions such as in RA [79], SLE [80], diabetes [73] and cardiovascular disease [81]. In RA, it was elegantly demonstrated that platelet activation is primarily a collagen-dependent articular process (via GPVI) leading to a high intra-articular PMP production that disseminates platelet-derived cytokines in the arthritic joint causing inflammation [79]. These findings provide evidence that PMPs play an amplifying role in the pathophysiology of RA [79]. In ITP, elevated PMP counts have been described earlier [82], however, PMPs in ITP have been mainly studied regarding their hemostatic role [82,83]. It has been suggested, in both newly diagnosed and chronic adult and childhood ITP, that high PMP levels may be associated with a lower bleeding tendency [82,83], with the highest level of PMPs being observed in newly diagnosed pediatric ITP patients [83]. Interestingly, a recent study demonstrated that plasma circulating PMPs are able to penetrate the BM during inflammation and interact with and reprogram BM-megakaryocytes in vitro and in vivo to enhance megakaryopoiesis [84]. It has not been established through what mechanism the PMPs are driven from the circulation to the BM, and which PMP-cargo is required to stimulate megakaryopoiesis. In line with this finding, another study also reported that human MK-EVs are able to induce *de novo* platelet production in WT mice [85]. It can be hypothesized that despite increased numbers of PMPs in ITP, their cargo is altered, which prevents them from functionally reprogramming BM-megakaryocytes to stimulate megakaryopoiesis and consequently platelet production. It would be interesting to see if therapeutic responses in ITP may perhaps decrease PMP quantity in plasma and through restored functional interaction with BM-megakaryocytes may stimulate platelet production. Additionally, it would be interesting as well to monitor specific PMP cargo in ITP to see whether this is altered over time due to specific therapeutic interventions. It has been demonstrated that PMP cargo can include microRNAs (miRNAs) which are able to infiltrate endothelial cells and reprogram macrophage gene expression and function [86]. In cardiovascular disease, it has been shown that circulating PMPs carry specific miRNAs and those PMP miRNAs are associated with disease activity [87]. A study identified 23 circulating miRNAs (which may be packed into PMPs) that differed between ITP patients (*n* = 10; 4/10 refractory and 6/10 newly diagnosed ITP) and healthy controls (HCs; *n* = 6) [88]. These observations included nine upregulated miRNAs in newly diagnosed ITP compared to HCs and a downregulation of three (miRNA-144-3p, miRNA-1281 and miRNA-3162-3p) out of those nine miRNAs in patients not responding to splenectomy or patients with a relapse requiring continuous therapy (refractory patients). Furthermore, this study examined the effect of four weeks of steroid treatment on circulating miRNAs in patients with ITP (*n* = 12), in some cases (4/12) combined with immune suppressants [88]. Patients in various disease phases were included (newly diagnosed *n* = 6, persistent *n* = 2, chronic *n* = 4). Patients with a platelet count >30 × 10^9^/L or a doubling of baseline platelet count and absence of bleeding symptoms, defined as responders (6/12, 50%), showed increased levels of plasma miRNA-320c after treatment [88]. And non-responders showed a decrease of miRNA-144-3p, miR-3141, miRNA-3162-3p and miRNA-4499 and increase of miRNA-1275 and miRNA-6126 after treatment with steroids [88]. This suggests that the levels of circulating miRNAs could be potential biomarkers to monitor therapy efficacy of steroids. In line with these results, levels of circulating miRNAs were analyzed in patients with primary ITP after treatment with TPO-RAs (romiplostim or eltromboplag) over 12 weeks [89]. The authors profiled miRNAs in 8 ITP patients and 8 HCs before treatment, and after two, six and twelve weeks of treatment, and found that during TPO-RA treatment 14 out of 81 evaluated miRNAs showed significant changes, with two of them being increased in ITP (miRNA-33a-5p and miRNA-195-5p) and one of them being decreased in ITP (miRNA-199a-5p) compared to HCs prior to treatment [89]. Furthermore, it was demonstrated that levels of miRNAs correlated with platelet counts at six weeks, which was particularly evident in patients who achieved complete response, suggesting that miRNAs may perhaps help in predicting response to TPO-RA treatment [89]. On a more functional level it has been shown that PMPs are able to inhibit the differentiation of highly purified human peripheral CD4+ CD25hi CD27+ CD127low/-FoxP3+ Tregs, by binding to Tregs in a P-selectin and partially CXCR3-dependent manner [90], supporting a contribution of PMP in impairing Treg function. Although this has not been investigated in ITP, it would be interesting to study if PMPs in ITP patients are indeed able to inhibit Treg differentiation, and if this process may be reversed in response to specific therapies. In summary, PMP-analyses, both quantitatively as well as functionally, warrants further investigations which may yield promising results for potential predictive utility of therapeutic responses in ITP. 

## 10. Future Recommendations

**Figure 1:**
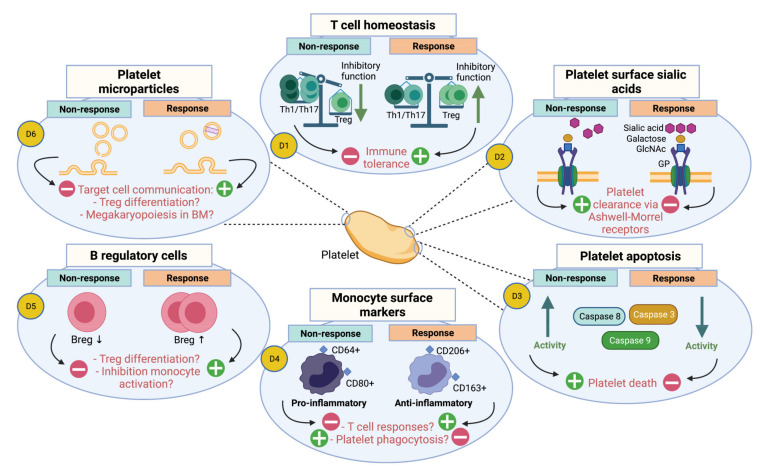
Potential diagnostic approaches for assessment of therapeutic responses in ITP. Promising diagnostic approaches for prediction of platelet count responses and thereby distinguish therapeutic responders from non-responders are depicted: T cell homeostasis (D1), Platelet surface sialic acids (D2), Platelet apoptosis (D3), Monocyte surface markers (D4), B regulatory cells (D5) and Platelet microparticles (PMPs). Each approach indicates therapeutic non-response vs. response, with their effects (+ indicates a stimulatory effect and - an inhibitory effect) on the features of the ITP pathophysiology. So far, the initial preliminary evidence may support the involvement of the following therapeutic modalities in relation to the diagnostic approaches: (D1) glucocorticosteroids, high-dose dexamethasone, IVIg, low-dose decitabine, TPO-RA; (D2) Corticosteroids, IVIg, TPO-RA; (D3) IVIg; (D4) glucocorticosteroids; (D5) TPO-RA; (D6) Corticosteroids, TPO-RA. This figure was created with BioRender.com.

Several therapeutic options are available for ITP, however, therapeutic responses are challenging to predict, due to heterogeneity of the ITP pathophysiology and the different stages of the disease. It will be important to mechanistically better understand the heterogeneity responsible for therapeutic relapse and refractoriness. In that respect, we recommend further research into exploitation of immune functions and platelet characteristics, which may assist in upfront discrimination of therapeutic responders from non-responders. Promising avenues to explore in particular include evaluation of T cell homeostasis. Additionally, evaluation of platelet surface sialic acids, platelet apoptosis, monocyte surface markers, B regulatory cells and platelet microparticles may have promising potential for prediction of therapeutic responses (see Figure 1 and Table 1). Not all therapeutic modalities may affect the aforementioned parameters, and it will be important to further investigate this. For this it will be important to take the phase of the disease into account, as newly diagnosed ITP patients may have different underlying pathophysiology than chronic ITP patients, which may differently affect therapeutic responses. In addition, it is imperative to also standardize clinical outcome measurements in both pediatric and adult ITP, as a high level of heterogeneity has been observed in outcome measurements across different studies in the past decade (e.g., definitions of platelet response, remission, bleeding and HRQoL) [91]. Furthermore, evaluation of immune characteristics would be of greater value if not only quantified during therapy but also validated on a functional level, including signaling pathways, to confirm their biological significance. The fact that an abnormality (e.g., low Tregs, etc.) is found during active disease and is normalized with treatment is not by itself a demonstration that such a mechanism is pathogenic. Thus, the direct link between ITP pathogenicity and altered immune parameters should be further explored and additional discussion regarding this is beyond the scope of this paper. Ultimately, it has been hypothesized that using a combination of therapeutic modalities with different modes of action will work better in refractory ITP patients than single-agent therapy [7]. While this should be further explored, it will be equally important to assess the impact of individual therapies on immune parameters as this will be an important first step in gaining insights into therapeutic responses which may allow timely adjustment of the therapeutic strategy. This may eventually facilitate individualized therapeutic management in ITP patients.

## Figures and Tables

**Table 1:** Immune features and potential initial diagnostic evaluations for monitoring and predicting therapeutic responses in ITP.

Immune Feature	Type of Therapy	Initial Diagnostic Methods	References
**T cell homeostasis**	Glucocorticosteroids High-dose dexamethasone Intravenous immunoglobulins (IVIg) Low-dose decitabine TPO-receptor agonist (TPO-RA)	Flow cytometry for quantification of CD4+ CD25+ FoxP3+ T regulatory cells (Tregs) and Th1 and Th17 cells, from peripheral blood.	[51,52,53,55]
**Platelet surface sialic acids**	Corticosteroids IVIg TPO-RA	Determination of platelet surface desialylation: platelet surface β-galactose in platelet-rich plasma by flow cytometry with labeled *Ricinus communis agglutinin* (RCA). binding of *Wheat germ agglutinin* (WGA) to determine N-acetylglucosaminyl residue (GluNAc) exposure. More WGA binding indicates a higher degree of desialylation.	[54,62,63,64]
**Platelet apoptosis**	IVIg	Detection of caspase-3, caspase-8 and caspase-9 proteins by western blotting or flow cytometry. Measurement of inner membrane potential (ΔΨm) by flow cytometry or Tetramethylrhodamine, ethyl ester (TMRE) fluorescence.	[70]
**Monocyte surface markers**	Glucocorticosteroids	Phenotyping I-MCs from peripheral blood mononuclear cells with flow cytometry using antibodies to CD14, CD16, CD64, CD80, CD163 and CD206.	[75]
**B regulatory cells**	TPO-RA	Flow cytometry to quantify CD19+ CD24hi CD38hi B regulatory cells (Bregs) in peripheral blood.	[24,54]
**Platelet microparticles**	Corticosteroids? TPO-RA?	Quantitative analysis of platelet microparticles (PMPs) in plasma: Flow cytometry using quantification and size-calibration microbeads and with use of CD41 labelling Super resolution microscopy Single molecular localization microscopy (SMLM) Structured illumination microscopy (SIM) Size exclusion chromatography	[88,92,93,94]
